# The engulfasome in *C. difficile*: Variations on protein machineries

**DOI:** 10.1016/j.anaerobe.2019.102091

**Published:** 2019-12

**Authors:** Abigail Kelly, Paula S. Salgado

**Affiliations:** Institute for Cell and Molecular Biosciences, Faculty of Medical Sciences, Newcastle University, Newcastle upon Tyne, UK

**Keywords:** *C. difficile*, *B. subtilis*, Sporulation, Engulfment, DMP, Q:AH

## Abstract

*Clostridioides difficile* infection (CDI) continues to be a substantial healthcare burden, and the changing disease profile raises new challenges in CDI management, both in clinical settings and in the community. CDI is transmitted by spores, which are formed by a subset of the cell population where an asymmetric septum is formed. A full copy of the chromosome is transported into the smaller compartment which is then engulfed by the mother cell. After engulfment, multiple metabolic and morphological changes occur, eventually resulting in the release of the mature spore. Whilst studies in the model organism *Bacillus subtilis* have demonstrated the importance of the DMP and Q:AH machineries in engulfment, it is becoming clear that there are fundamental differences in the way the two organisms organise these machineries. As spores are the infectious agent in CDI, it is crucial to understand how these dormant cells are formed, and how sporulation can be prevented or disrupted with the view of reducing CDI. Here, we review the current literature on the DMP and Q:AH machineries in *C. difficile*, and how they compare and contrast to those of *B. subtilis*.

## Highlights

• Overview of the DMP and Q:AH engulfment machineries in *C. difficile*.
• Analyses of the conservation of DMP across Bacilli, Clostridia and other bacteria.
• Proposes a multi-protein complex required for engulfment: the engulfasome.
• Highlights differential arrangements of engulfasome in *B. subtilis* and *C. difficile*.

## 1 Introduction

*Clostridioides difficile*, commonly known as *Clostridium difficile,* is a Gram-positive anaerobic human pathogen capable of causing disease ranging from mild, self-limiting diarrhoea to severe pseudomembranous colitis and death. It is estimated that there are over 150,000 new cases of *C. difficile* infection (CDI) each year in Europe [1], with an associated cost of €3 billion per year [2]. With the recently described shift in disease profile, from affecting hospitalised patients treated with broad spectrum antibiotics towards community-associated “antibiotic naïve” individuals, and an increasingly ageing population, CDI incidence is expected to double over the next 4 decades [[2], [3], [4]]. This reinforces the requirement for a greater understanding of the basic biology of this organism.

**Fig. 1:**
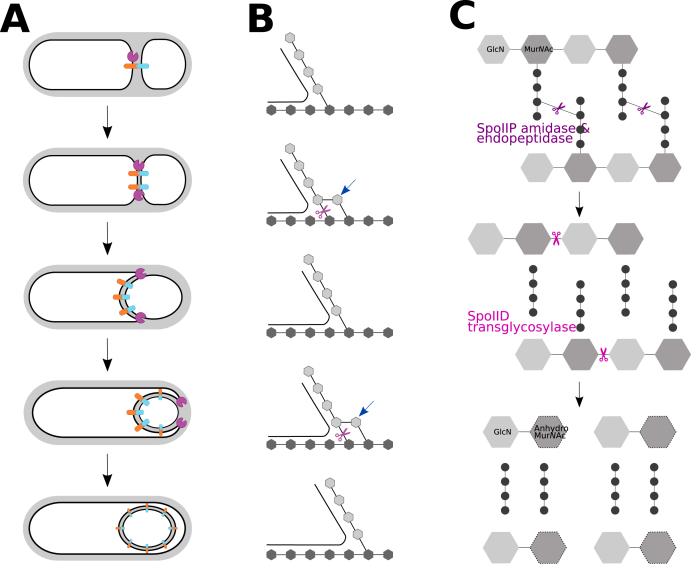
Molecular basis of engulfment. Sporulation requires the coordinated activity of the DMP machinery. A) The larger mother cell first forms an asymmetric septum to which the DMP (purple) and Q:AH (orange and blue, respectively) machineries are recruited. These proteins then track the advancing membrane as the mother cell engulfs the forespore, eventually forming a double membraned protoplast. Once formed, the mature spore is then released by mother cell lysis. B) Model for peptidoglycan remodelling during engulfment [22]. Forespore peptidoglycan (light grey) and mother cell peptidoglycan (dark grey) are used as a template for coordinated insertion (blue arrow) and digestion (purple scissors) of newly inserted peptidoglycan. This creates the space for the mother cell membrane to move into, driving engulfment. C) Enzymatic activity of SpoIIP and SpoIID. Alternating GlcN (grey) and Mur*N*Ac (dark grey) residues form glycan strands crosslinked by short peptide stems in *C. difficile* peptidoglycan. The amidase activity of SpoIIP (purple) cleaves the peptide stems from the glycan strands and its endopeptidase activity breaks the crosslinks between the stems, resulting in long “denuded” glycan strands. These glycan strands are the substrate for SpoIID (magenta) lytic transglycosylase activity, resulting in the formation of 1,6- anhydroMur*N*Ac termini. (For interpretation of the references to colour in this figure legend, the reader is referred to the Web version of this article).

To allow transmission between hosts and persistence in the aerobic environment, *C. difficile* produces endospores (herein referred to as spores). During sporulation, the vegetative cell produces an asymmetric septum at one cell pole, creating a smaller compartment. The larger mother cell then engulfs the forespore to form a cell-within-a-cell structure, which then matures into the spore. Once the mature spore is formed, the mother cell lyses, releasing the spore into the environment (Fig. 1A) [5,6].

Engulfment requires the coordinated, targeted synthesis and degradation of peptidoglycan (PG) at the leading edge of the engulfing membrane to allow the mother cell to completely engulf the forespore (Fig. 1) [7]. SpoIID, SpoIIM and SpoIIP have been identified as a protein complex (DMP machinery) involved in this targeted peptidoglycan degradation [[8], [9], [10], [11], [12], [13], [14], [15]]. Two further proteins are thought to be key in engulfment: the mother cell-produced SpoIIIAH and the forespore-produced SpoIIQ. These proteins interact across the intermembrane space, though the function of the SpoIIQ:SpoIIIAH (Q:AH) complex is so far undetermined, with several hypotheses currently proposed [16].

Characterisation of the DMP and Q:AH complexes has primarily been carried out in the Gram-positive model organism *Bacillus subtilis*. SpoIIIAH is part of the *spoIIIA* operon containing 8 genes – *spoIIIAA* to *spoIIIAH* – which are thought to form a complex with SpoIIQ (the A-Q complex), required for engulfment in *B. subtilis*. Recent investigations of these machineries in *C. difficile* have highlighted that the two organisms do not necessarily use the same protein components in the same ways.

## 2 Peptidoglycan remodelling – D(M)P machinery

The DMP machinery is not unique to *B. subtilis* and *C. difficile* but is found across endospore-formers [17,18]. In order to probe conservation of the three proteins in more detail, we performed different Hidden Markov Model (HMM) searches: within a set of representative endospore-formers [19] (Table S1), across all Bacilli and all Clostridia, as well as all bacteria. As expected, all three proteins were found in the representative endospore-formers apart from absence of orthologues of SpoIIP in *Symbiobacterium thermophilum* and SpoIIM in *Lysinibacillus sphaericus* (Table S1). Protein sequence alignments show that, while the full-length SpoIIM is well conserved across these endospore-formers, SpoIID and SpoIIP conservation is restricted to the core enzymatic domains (Pfam domains PF08486 and PF07454, respectively), with poor conservation at the N-terminus (Fig. S1). Interestingly, SpoIIP is less well conserved across clostridial representatives, with several insertions/deletions within the Pfam domain that could reflect varying enzymatic activities and/or substrate specificity. Alignment of the representative Bacilli and Clostridia proteins allowed us to generate a HMM profile for each protein. Searching for orthologues within each class readily identified DMP orthologues. When expanding this search to all bacteria, members of the SpoIID/LytB family and peptidoglycan hydrolases were readily identified across all phyla. Analysis of the orthologues retrieved in this wide search revealed several variations in domain architecture, with the canonical SpoIID domain architecture, as found in *B.* subtilis, clustering to spore-forming bacteria such as the Firmicutes. Conversely, SpoIIM and SpoIIP orthologues identified using our HMM profiles to search across all bacteria were restricted to the Firmicutes. In this case, the orthologues identified contained more conserved domain architectures, suggesting that the DMP proteins represent a machinery specific to endospore-formers in this phylum.

### 2.1 SpoIIP

In the model organism *B. subtilis*, SpoIIP production is under the transcriptional control of the RNA polymerase sigma factor σ^E^, which in turn is controlled by the master regulator Spo0A [5]. Whilst *C. difficile* SpoIIP production is also ultimately under the control of Spo0A, it is directly under the control of σ^F^ [20,21]. As σ^E^ controls gene transcription in the mother cell and σ^F^ in the forespore, this divergent transcriptional control implies that the DMP machineries themselves are differentially organised: while *B. subtilis* SpoIIP (*Bs*IIP) is thought to be anchored to the mother cell membrane, *C. difficile* SpoIIP (*Cd*IIP) would be on the forespore side of the double membrane system.

In further contrast to the current *B. subtilis* model, Ribis *et al*. (2018) propose that SpoIIP may not necessarily be anchored to the forespore membrane throughout engulfment. Instead, they suggest that SpoIIP can also be cleaved post-translationally, as they detect the presence of 3 isoforms: full length (∼38 kDa), truncated (∼36 kDa) and cleaved forms (∼30 kDa) of SpoIIP on western blots. Interestingly, the band corresponding to this cleaved form is not seen in *sigE* and *sigG* Clostron mutants [21], suggesting that cleavage only happens once engulfment has been completed and could therefore be a mechanism of SpoIIP post-engulfment clearance. Alternatively, it is possible that cleaved SpoIIP is an active isoform that is released into the intramembrane space allowing free access to peptidoglycan. This could relate to the hypothesis originally proposed in *B. subtilis* by Morlot *et al*. (2010): once SpoIIP has acted as an endopeptidase and amidase, it is released from the peptidoglycan and associates with the next “free” binding site in the glycan chain. Perhaps access to the next free site involves truncation of SpoIIP in *C. difficile*, generating the isoform observed by Ribis *et al*. However, it is unclear how a “free” SpoIIP would ensure coordinated activity with SpoIID, as proposed in the current engulfment model [22]. Interestingly, Dembek and colleagues detected the same 3 isoforms in the membrane associated fraction, but only bands corresponding to full length and truncated SpoIIP in the soluble fraction, which suggests that this cleaved form is still somehow associated with the membrane [15].

Detailed analysis of predicted signal peptides and transmembrane helices in *B. subtilis* and *C. difficile*, undertaken to help understand the potential localisation of SpoIIP and the relevance of the different isoforms, revealed unexpected results: while *Bs*IIP is predicted to have an N-terminal transmembrane helix (residues 1–19, TMHM [23]), neither SignalP 5.0 [24] nor Phobius [25] predict the presence of a signal peptide. Conversely, *Cd*IIP is predicted to have a signal peptide (residues 1–25, Phobius and SignalP 5.0) but not a transmembrane helix. Although the lack of identification of the signal peptide in *B. subtilis* could relate to the absence of the canonical AxA type I peptidase cleavage site [26], the prediction that *Cd*IIP does not contain a transmembrane helix could explain the presence of the 3 isoforms detected by Ribis *et al*. (2018). If SpoIIP is not directly tethered to the membrane but associated with it via interaction(s) with other protein(s), it could be more prone to degradation and/or cleavage, resulting in the appearance of a cleaved form. Whether SpoIIP is embedded in the membrane or interacting with membrane-bound proteins such as SpoIID, SpoIIM or others is worth investigating further.

In *B. subtilis,* GFP-fusion constructs have demonstrated that SpoIIP initially localises to the midpoint of the septum, then follows the advancing membrane as the mother cell engulfs the forespore [9,14]. Investigations into SpoIIP localisation in *C. difficile*, especially higher resolution microscopy techniques, may clarify if the isoform(s) localise to the forespore or mother cell membrane, or if they are present in the periplasm as free isoforms. This in turn would inform our current model of DMP organisation at the advancing membrane of *C. difficile*.

In both *C. difficile* and *B. subtilis*, strains lacking *spoIIP* do not progress beyond the formation of the asymmetric septum and are unable to produce heat-resistant spores [15,27], consistent with SpoIIP being the first of the DMP complex to act enzymatically. *B. subtilis* Δ*spoIIP* cells show characteristic membrane bulges, which are thought to be the consequence of uncoupling of peptidoglycan degradation and formation [7], combined with chromosome translocation to the forespore by SpoIIIE [28]. Alternatively, these could indicate a collapse of the double membrane system surrounding the forespore [29]. Strikingly, in *C. difficile* these membrane bulges are absent [15], perhaps suggesting a degree of redundancy that is able to compensate for the loss of SpoIIP-mediated peptidoglycan digestion that is absent in *B. subtilis*. The more severe phenotype observed in *C. difficile*, where sporulating cells are arrested soon after asymmetric septa formation, may also indicate that the absence of SpoIIP prevents any further PG remodelling and/or synthesis.

Whilst membrane bulges are absent in *C. difficile* Δ*spoIIP* mutants, a “bearding” phenotype is observed: the spore coat is mislocalised, not adhering to the forespore, but sloughing off [21]. This bearding phenotype was also seen in Δ*spoIIQ* mutants and, to a lesser extent, in Δ*spoIID* cells. Interestingly, Δ*spoIIM* cells that failed to complete engulfment showed coat bearding [21]. Deletion of both *spoIIQ* and *spoIID* resulted in a bearding phenotype equivalent to the *spoIIP* single mutant. This suggests that the DP/Q:AH machinery of *C. difficile* may have an additional role, either directly or indirectly, in the correct localisation of the spore coat.

Based on sequence similarity with *B. subtilis* SpoIIP and the amidase CwlV [9], several residues are proposed to be involved in amidase activity in *Cd*IIP: two catalytic histidine residues, H142 and H222, and a conserved glutamate, E309. When peptidoglycan is incubated with H142R or H222R mutants of *Cd*IIP, no denuded glycan strands were produced and these mutants were unable to restore the sporulation defect in Δ*spoIIP* cells, indicating that they are essential for protein function [15]. Complementation with a *Cd*IIP E309A mutant also failed to restore heat-resistant spore production in Δ*spoIIP* cells [21] but the direct effect of this mutation on enzymatic activity has not been tested.

**Fig. 2:**
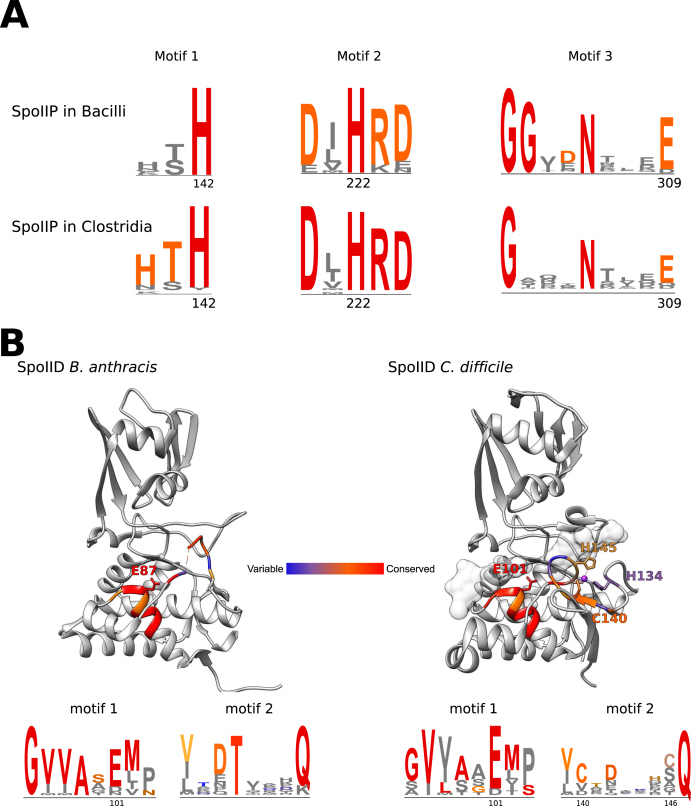
Conservation of SpoIIP and SpoIID A) The three motifs containing the three histidines and the glutamate required for SpoIIP amidase activity are shown, highlighting the conservation of the catalytic residues. While the histidines are strictly conserved, the glutamate is present in most organisms, but substituted to aspartate in some cases in both Bacilli and Clostridia. Other features vary between the two bacterial families: the first histidine in motif 1 is well conserved only in Clostridia, whilst the double glycine feature is only found in Bacilli. These differences could relate to variations in specificity and/or protein stability. B) Structures of SpoIID from *B. anthracis* (PDB ID 4RWR, left) and *C. difficile* (PDB ID 5I1T, right) with motifs 1 and 2 coloured according to conservation as highlighted in the corresponding web logos. Whilst motif 1, which contains the catalytic glutamate, is well conserved in both bacterial families, motif 2 is poorly conserved, with the zinc-binding residues only present in the clostridial proteins.

Notably, both catalytic histidines are strictly conserved across all Bacilli and Clostridia orthologues identified using our HMM profiles and the glutamate is highly conserved, with functional substitution with aspartate seen in a small fraction of bacilli or clostridial SpoIIP orthologues (Fig. 2A). Analysis of the regions around these residues allowed us to identify three possible motifs: HT**H**, containing H142 (motif 1), Dx**H**RD defining the region surrounding H222 (motif 2), and GgxxNxxx**E** (motif 3) containing E309 (Fig. 2A). Although the general motifs are conserved, some differences are seen between Bacilli and Clostridia, which might be reflected in enzymatic activity and/or specificity in different bacteria. Motif 2 is the most conserved, with all residues strictly conserved in clostridial SpoIIP and well conserved in the bacilli orthologues (Fig. 2A). The first histidine in motif 1 is only conserved in the clostridial proteins, which raises the interesting possibility of some level of specificity surrounding this motif. Conversely, whilst the first glycine and the asparagine in motif 3 are strictly conserved, the second glycine is only conserved in the bacilli proteins, again suggesting potential divergent specificity between the two families of proteins. It will be interesting to investigate the role of other residues in these motifs in SpoIIP activity and/or specificity. Determination of the structure of SpoIIP, particularly in complex with different substrates, would also provide insight into the mechanism of catalysis and the role of each proposed catalytic residue.

Interestingly, CwlV has been shown to preferentially require zinc for activity, though it is also able to use manganese and cobalt, but not magnesium or calcium [30]. However, analysis of the metal content of *Cd*IIP by inductively coupled plasma mass spectrometry (ICP-MS) did not detect any of the metals tested, despite the enzyme being catalytically active [15]. Further investigation is needed to reveal the enzymatic mechanisms of the dual activity of SpoIIP: can amidase activity occur independently of endopeptidase activity or *vice versa*?

### 2.2 SpoIID

In both *C. difficile* and *B. subtilis*, *spoIID* is under the direct control of the mother cell sigma factor, σ^E^ [20,21,31] suggesting it is produced on the mother cell side of the membrane. GFP-fusions demonstrate SpoIID initially localises to the septal midpoint in *B. subtilis*, then follows the advancing membrane throughout engulfment [9], though this has yet to be demonstrated in *C. difficile*.

SpoIID is a lytic transglycosylase that produces muropeptides with terminal 1,6-anhydro Mur*N*Ac residues, but it requires “denuded” peptidoglycan*, i.e*. the products of SpoIIP activity [10,11,15]. It has been speculated that the sequential activity of SpoIIP followed by SpoIID prevents the release of muropeptides from the peptidoglycan sacculus, which can induce spore germination [32]. Indeed, high pressure liquid chromatography mass spectrometry (LC-MS) analysis of PG digestion by SpoIID showed no enzymatic activity in the absence of SpoIIP [15], confirming the earlier conclusions of Nocadello *et al*. (2016) using dye release-based assays.

Due to the overall sequence similarity between *B. subtilis* SpoIID and LytB, it has been suggested that SpoIID may have a stimulatory effect on the amidase activity of SpoIIP, in a manner similar to the stimulatory effect of LytB on LytC [9]. Using *B. subtilis* SpoIID and *E. coli* peptidoglycan in a dye release assay, Morlot *et al*. (2010) demonstrated stimulation of SpoIIP activity by catalytically inactive SpoIID mutants. However, this was not replicated in semi-quantitative LC-MS experiments where *E. coli* peptidoglycan was digested with *Cd*IIP in the presence of inactive *C. difficile* SpoIID (*Cd*IID) mutants [15]. This could indicate a less intrinsically controlled PG hydrolysis but the observed differences could reflect the increased sensitivity and accuracy of the LC-MS method, rather than a functional divergence between the DMP complexes of *B. subtilis* and *C. difficile*. Future studies towards understanding of the interplay between the two enzymes in both organisms should rely on similar methodology when assessing activity to avoid ambiguity.

Deletion of *spoIID* in *C. difficile* prevents sporulation but still allows the formation of an asymmetric septum and membrane curvature. This is consistent with the activity of SpoIID only occurring following the activity of SpoIIP as SpoIIP depleted cells are unable to form curved membranes.

SpoIIP activity where SpoIID is absent can lead to asymmetric engulfment [21], as is observed in *B. subtilis spoIID* mutants [13]. When double Δ*spoIID*/Δ*spoIIP C. difficile* mutants were complemented with catalytically inactive *spoIIP*_*E309A*_, the asymmetric engulfment phenotype was alleviated, whereas complementation of the double mutant with catalytically inactive *spoIID*_*E101A*_ resulted in asymmetry comparable to that seen in the Δ*spoIID* mutant [21]. This indicates that SpoIIP activity is somehow implicated in asymmetric engulfment in the absence of SpoIID activity in *C. difficile* [21].

Multiple structures of SpoIID [11] are available in the Protein Database (PDB): *B. anthracis* (PDB ID: 4RWR), *C. difficile* in apo conformation (PDB ID: 5TXU) and *C. difficile* in complex with triacetylchitotriose (NAG_3_) (PDB ID: 5I1T). SpoIID consists of an α-helix-rich “hand” domain and a β-strand-rich “arm” domain, with the substrate bound in the hand domain [11]. Various experiments have demonstrated E101 to be the major catalytic residue of *Cd*IID, with peptidoglycan digestion experiments showing abrogation of activity *in vitro* [11,15], and sporulation efficiency assays demonstrating an inability to produce heat-resistant spores [15]. The NAG_3_ substrate found in the *C. difficile* structure (5I1T) makes direct contacts with SpoIID across the subsites of the binding region. Several residues are responsible for interactions with the acetyl group of NAG_3_ at different subsites. Importantly, of these, only Y194, which is implicated in the correct orientation of the sugar for catalysis, abrogates activity [11]. Therefore, the importance for activity of the interactions between SpoIID and the acetyl groups, as observed in the crystal structure, is still unknown. Indeed, *C. difficile* peptidoglycan, although synthesised as Glc*N*Ac, is predominately deacetylated to glucosamine (Glc*N*) once incorporated into vegetative cells [33], and therefore the acetyl groups present in NAG_3_ would likely be absent during SpoIID activity, so the biological relevance of these interactions is unclear.

*Cd*IID can coordinate zinc via C140, C146, H145 and H134 as proposed in the structure [11] and confirmed by ICP-MS [15]. Mutating these residues to alanine both *in vivo* and *in vitro* revealed important differences: C140A mutant is not active and cannot rescue the sporulation phenotype, whilst mutations of C146 and H134 do not completely prevent zinc binding [15] and have limited to no effect on sporulation efficiency, despite somewhat reduced activity *in vitro* [11,15]. Finally, mutation of H145 prevents zinc binding and peptidoglycan degradation by SpoIID, but has limited effect on sporulation efficiency [15]. These results suggest that either: i) not all zinc binding residues are necessary for enzymatic activity or protein stability, ii) zinc is not required for catalysis or iii) there is an as-yet unidentified mechanism that can at least partially compensate for the absence of zinc. Further investigation into the crystal structures of SpoIID carrying these point mutations, as well as in complex with biologically relevant substrates, would provide more information on the enzymatic mechanism.

Our analysis of SpoIID conservation across other bacteria may also inform future investigations. The catalytic glutamate (E101 in *Cd*IID) is strictly conserved in all Bacilli and highly conserved in clostridial species (Fig. 2B). A motif can be defined surrounding this residue – GVVxxEMP/S – which is generally conserved across all species. Conversely, the cysteine-rich region containing the residues that coordinate zinc, identified as yCxdxxHCQ, is poorly conserved, even within the Clostridia, and is absent in Bacilli (Fig. 2B). Interestingly, the most conserved residue in the representative Clostridia is the first cysteine (C140 in *Cd*IID), which is the only residue showing a clear phenotype in *C. difficile* [15].

### 2.3 SpoIIM

SpoIIM is predicted to have 5 transmembrane helices, with the N-terminal facing the cytoplasm and an extracellular C-terminal. SpoIIM orthologues are restricted to the Firmicutes, suggesting this is part of a specific adaptation required for sporulation. SpoIIM is well conserved across the core transmembrane helices, with few insertions or deletions, suggesting that the number of helices and protein topology are relevant (Fig. S1).

In *B. subtilis*, expression of SpoIIM is regulated by σ^E^ [8] and the protein is embedded in the mother cell membrane at the asymmetric septa, forming foci at the edges of the advancing membrane during engulfment, although a faint signal of GFP-SpoIIM fusion was also detected in the cytoplasmic membrane [10,14]. Whilst SpoIIM is not predicted to have any enzymatic activity, it is the first to be recruited to the mother cell membrane during sporulation, and knockout of *spoIIM* prevents sporulation in *B. subtilis* [8,9]. Several factors have been proposed to contribute to this effect: SpoIIM is thought to recruit SpoIID and SpoIIP in *B. subtilis* [9], it could be required for the synthesis of σ^G^-associated RNA polymerase dependent genes [8] or partly because of its effect on A-Q complex activity [34]. GFP fusions in *B. subtilis* demonstrated that SpoIIP localisation to the membrane at the leading edge requires SpoIIM: in the absence of SpoIIM, both SpoIIP and SpoIID mislocalised to the mother cell cytoplasm [9]. Lack of SpoIIP also prevented SpoIID localisation, demonstrating the hierarchical recruitment of the DMP machinery to the leading edge [9].

Interestingly, this does not appear to be the case in *C. difficile* as SpoIIM seems to be dispensable for sporulation: deletion of *spoIIM* showed no difference between the number of cells completing engulfment and forming mature spores in comparison to wild type [15,21]. Surprisingly, expression of SpoIIM in *C. difficile* seems to be directly dependent on Spo0A transcription, not on either σ^E^ or σ^F^ [21,35,36], which suggests the protein could be expressed in both the forespore and mother cell compartments. These observations indicate that SpoIIM is playing different roles in *B. subtilis* and *C. difficile*, demonstrating the differences between the machineries in the two organisms.

Recent bacterial adenyl cyclase two hybrid (BACTH) system work has demonstrated that *C. difficile* SpoIID and SpoIIP interact without the requirement for SpoIIM [15]. In this context, it is intriguing that SpoIIM is so well conserved amongst endospore-formers, which would suggest an important role in sporulation. Perhaps *C. difficile* has a mechanism for SpoIID-SpoIIP interaction and localisation that is independent of SpoIIM, or perhaps there are redundancies within other components of the sporulation machineries that can compensate for the loss of SpoIIM in *C. difficile* which are absent in *B. subtilis*.

Such redundancy may come partially from the SpoIIQ: SpoIIIAH complex, which behaves differently in *C. difficile* [37,38].

## 3 Forespore – mother cell communication - Q:AH complex

A key signature of endospore-formers is the presence of the forespore gene *spoIIQ* [39] and the mother cell *spoIIIA* operon, composed of the *spoIIIAA to spoIIIAH* genes [17,18]. The eight proteins from the *spoIIIA* operon are proposed to form a complex at the mother cell membrane. Interactions between SpoIIIAH and SpoIIQ observed in both *B. subtilis* [[40], [41], [42], [43], [44]] and *C. difficile* [37,38] would then link the mother cell and forespore membranes via the so called A-Q complex, reviewed recently by Morlot and Rodrigues (2018). Focus here will be on recent studies of these proteins in *C. difficile* and their interplay with the DMP machinery, highlighting key differences in comparison to *B. subtilis*.

### 3.1 SpoIIIAH

SpoIIIAH is expressed in early engulfment under the control of σ^E^ in the mother cell [45] and is required for efficient sporulation [46]. In *C. difficile* it seems to be more crucial, as cells lacking *spoIIIAH* were arrested at early engulfment with both membrane collapse and inverted septa observed in Δ*spoIIIAH* cells that had begun sporulation [37,38]. These observations strengthen the hypothesis that SpoIIIAH, and by extension SpoIIQ, might be important for integrity of the double membrane system during engulfment in *C. difficile*.

Absence of SpoIIIAH also leads to detachment of the coat from the forespore and/or mislocalisation to the mother cell cytosol [37]. Whether this is due to instability of the double membrane system failing to properly localise the coat proteins or direct interactions with SpoIIIAH remains to be fully investigated [37,38,46].

Using an innovative split-SNAP approach, Serrano and colleagues (2016), demonstrated that, in *C. difficile*, SpoIIIAH and SpoIIQ interact *in vivo*, across the double membrane [38]. Interestingly, unlike in *B. subtilis* where correct localisation of SpoIIIAH at the septal membrane is dependent of SpoIIQ, *C. difficile* SpoIIIAH (*Cd*AH) seems to at least partially be able to localise at the septa independent of SpoIIQ [38]. It is possible that other redundant localisation mechanisms are present in *C. difficile,* namely the DMP machinery, which might be involved considering the direct interactions between SpoIIIAH and these proteins observed using BACTH assays [15]. Interestingly, localisation of SpoIIIAH in strains lacking *spoIID* and *spoIIP* seemed unaffected at the early stages of engulfment [15]. As these mutants in *C. difficile* are stalled soon after asymmetric division, particularly in the case of Δ*spoIIP*, it is unclear whether SpoIID or SpoIIP are involved in localisation of SpoIIIAH later in engulfment.

Although the exact role of SpoIIIAH and Q:AH complex is still to be determined, *Cd*AH seems to be important for later stages of sporulation as its absence severely compromises σ^G^ activity in the forespore and the mother cell-specific σ^K^ factor following engulfment completion [38]. As it has been shown that σ^K^ activity is partially independent of σ^G^ in *C. difficile* [35,47,48]) and considering that the Q:AH channel is degraded soon after engulfment completion in *B. subtilis* [41], this is surprising and hints at other roles for the Q:AH channel beyond connecting the mother cell and forespore during engulfment.

Although no structures are available for *C. difficile* SpoIIIAH, sequence similarity and structure predictions based on the available structures of the *B. subtilis* Q:AH heterodimer indicate that the Q:AH interface is highly conserved in *C. difficile* and other endospore-formers [39]. Structure determination of the *C. difficile* complex and, more importantly, of the full-length multimeric ring is essential to fully understand the function of SpoIIIAH and SpoIIQ.

### 3.2 SpoIIQ

SpoIIQ, produced under the control of σ^F^, is present at the forespore membrane in both *B. subtilis* [40,49] and *C. difficile* [35,38,47,48] and is required for sporulation [21]. Notably, *C. difficile* strains lacking *spoIIQ* show a more severe phenotype than similar mutants in *B. subtilis,* with most cells stalled at earlier stages of engulfment and bulging of the forespore double membrane towards the mother cell [38], a phenotype proposed to relate to lack of, or reduced, PG hydrolysis [13,27].

SpoIIQ proteins belong to the LytM domain family of endopeptidases, but whilst most Bacilli have a degenerate catalytic zinc-binding motif, the associated HxxxD and HxH motifs are highly conserved across Clostridia [39]. The bulging phenotype does not appear to relate to potential peptidoglycan degradation activity as mutations reverting the motif to that present in *B. subtilis* SpoIIQ (*Bs*Q) did not result in a bulging phenotype, although sporulation is still impaired [38]. This supports the model where Q:AH functions as a zipper for the double membrane system as proposed by Blaylock *et al*. (2004). The requirement for an intact LytM domain remains puzzling, but evidence suggests that zinc binding might be important for Q:AH complex stability [38] as the LytM motif mutant could not bind zinc and the complex formed with SpoIIIAH was less stable *in vitro* and *in vivo* [38]. This led to the hypothesis that the LytM motif and its zinc binding capability are important for structural stability of SpoIIQ and, importantly, the Q:AH interface in *C. difficile*.

Analysis of structural predictions based on the structure of the *B. subtilis* Q:AH heterodimer and sequence similarities indicates that the zinc binding region is close to the Q:AH interface. However, while the interaction interface of *Bs*Q involves a helix-strand-strand motif, this region is predicted to be mostly unstructured in *Cd*Q [38]. Moreover, proposed models of the multimeric Q:AH ring place this region at the interface between neighbouring Q:AH subunits. An elegant hypothesis would be that zinc binding anchors this loop region in a specific conformation that provides a more structurally stable interface for complex formation and/or stability.

Localisation of SpoIIQ in *C. difficile* seems to be dependent on SpoIIIAH and, in the very early stages of engulfment, SpoIIQ and SpoIIIAH localise to the asymmetric septum even in the absence of SpoIID or SpoIIP [15,38]. This is another example where the two protein machineries seem to differ between *C. difficile* and *B. subtilis,* since *Bs*Q localisation depends on SpoIIIAH, GerM and the enzymatic activities of SpoIID and SpoIIP [34,50,51]. Interestingly, it has been suggested that the LytM domain interacts with other mother cell protein(s) in *B. subtilis* that also contribute to the correct localisation of SpoIIQ [34]. LytM domains are required for peptidoglycan thinning in dividing cells and have been proposed to mediate interactions with PG [52]. Although *Bs*Q does not seem to interact with PG [34,53], an interesting hypothesis would be that, in *C. difficile*, the LytM domain interacts with the peptidoglycan, providing an alternative or redundant mechanism to anchor the Q:AH complex and the DP machinery throughout engulfment. Further investigation of the exact function of the LytM domain and an intact zinc-binding motif are required to elucidate the role of SpoIIQ and its complex partner SpoIIIAH.

Similarly to SpoIIIAH, SpoIIQ has also been shown to be involved in σ^G^ and, to a lesser extent, σ^K^ activity in the forespore and mother cell, respectively [38]. Further investigations of both the engulfment machinery and the activation of the sigma factors are required to fully understand the driving forces and regulation of engulfment and sporulation in *C. difficile.*

## 4 Additional proteins involved in engulfment

### 4.1 SpoIIIAA-AG

In *B. subtilis*, the *spoIIIA* operon proteins SpoIIIAA to SpoIIIAG form a complex with SpoIIIAH at the mother cell membrane [43] that, together with SpoIIQ, define an essential sporulation complex (A-Q). SpoIIIAA is an ATPase that has been proposed to power the activity of the A-Q complex to drive sporulation [43]. Investigations of the role of SpoIIIAA in *C. difficile* by the Shen group indicate that SpoIIIAA and probably AB to AF are only partially required for engulfment [37]. Indeed, strains where the *spoIIIA* operon is disrupted by mutating *spoIIIAA* could still complete engulfment in 10–20% of the cases but no heat-resistant spores were produced. These mutants still produced SpoIIIAH to normal levels, as expression of SpoIIIAH and SpoIIIAG is under control of a second, internal promoter [37]. Lack of SpoIIIAA causes the coat to detach from the forespore and/or mislocalise to the mother cell cytoplasm and bulging of the forespore membrane in a fraction of the cells [37]. It is important to note that forespore collapse has been described in the absence of SpoIIQ or the SpoIIIA components in similar experiments in *B. subtilis* [43]. However, the phenotype in *C. difficile* involves the membrane bulging towards the mother cell cytoplasm, rather than collapsing inwards towards the forespore cytoplasm. SpoIIIAA function in *C. difficile* engulfment seems to be reliant on ATP binding rather than hydrolysis, as mutations in the ATPase Walker A but not Walker B motifs affect effective engulfment and sporulation [37], unlike in *B. subtilis*, where both activities are essential [43].

*spoIIIAG* is expressed under the control of the same internal promoter as *spoIIIAH*, as well as the *spoIIIA* operon promoter in both *B. subtilis* [54] and *C. difficile* [20]. The protein is anchored to the mother cell membrane by its N-terminus and, similarly to SpoIIIAH, contains a ring-building motif (RBM) [44]. Cryo-EM structure determination has shown the formation of a 30-mer SpoIIIAG ring in a cup-and-saucer architecture with a unique structural motif insertion within the RBM forming most of the cup region [55]. SpoIIIAG ring formation is needed for Q:AH function in *B. subtilis* (Rodrigues et al., 2016) which has led to the suggestion that SpoIIIAG could stack against the SpoIIIAH ring that itself interacts with the putative SpoIIQ ring [16]. To date, the role of SpoIIIAG has not been investigated in *C. difficile,* so whether a similar interplay between the two *spoIIIA* operon proteins is required is still unknown.

## 5 Other proteins

Recent work has shown that other factors might interact and/or affect the Q:AH complex in *B. subtilis*: GerM [51] and SpoIIIL [56]. Absence of either protein results in small forespores with irregular membranes and limited transcriptional potential [51,56]. Initial models suggested that GerM might form a ring that stacks with the SpoIIIAG and SpoIIIAH rings [16], but recent structural characterisation of GerM did not reveal physiological relevant oligomerisation [57]. The structure of the two GerMN domains in GerM seem to be able to adopt an open and closed conformation and the presence of non-canonical RBMs could relate to the role of GerM in localising SpoIIQ [57]. However, GerM interaction with the AG/AH proteins and the mechanism that allows it to partially compensate for lack of AH remain to be determined. Importantly, no orthologues of GerM or SpoIIIL are present in *C. difficile,* which implies that a simplified version of the A-Q complex is present or that other as-yet unidentified mechanisms are involved in complex formation and/or function.

## 6 Discussion and outlook

### 6.1 The engulfasome

**Fig. 3:**
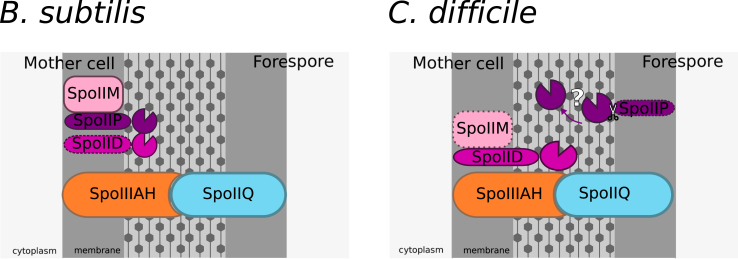
Current views of the engulfasome in *B. subtilis* and *C. difficile* In *B. subtilis*, SpoIIM (pink) recruits SpoIIP (purple) which in turn recruits SpoIID (magenta) to the mother cell membrane. This machinery is proposed to help recruit the Q:AH (blue and orange, respectively) to the septum and the leading edges of the membrane during engulfment. In *C. difficile*, SpoIIP is transcribed on the forespore side and recent work by Ribis et al. (2018) suggests *Cd*IIP may be proteolytically processed and not necessarily membrane anchored. SpoIIIAH (orange) and SpoIIQ (blue) interact across the intramembrane space holding the mother cell and forespore together. Direct interactions between *Cd*IID and *Cd*IIP with Q:AH suggest a multimeric complex, part of the engulfasome, that is required for engulfment. Identification of other proteins involved and the exact nature of the interactions within the engulfasome need to be investigated. (For interpretation of the references to colour in this figure legend, the reader is referred to the Web version of this article.)

Evidence suggests that an interplay between different machineries and proteins is required for effective engulfment in endospore-formers. We suggest that a holistic view of these different machineries and their intricate network of interactions and dependencies is necessary and suggest “engulfasome” as an encompassing term. The current known components of this machinery have different organisations in *B. subtilis* and *C. difficile*, as reviewed here (Fig. 3). We propose that other PG synthesis and degradation enzymes, as well as other yet unidentified regulating or assembly factors, would be part of the engulfasome and future research into these other potential components is essential for a complete understanding of the engulfment mechanism.

One of the most striking differences currently known between the engulfasome system in *C. difficile* and *B. subtilis* is the lack of absolute necessity for SpoIIM in *C. difficile* [15,21]. Considering both the direct interactions of SpoIID and SpoIIP demonstrated in *C. difficile* [15] and the transcriptional control of these two proteins in different cellular compartments - SpoIID: mother cell σ^E^; SpoIIP: forespore σ^F^ - [21] - it is tempting to hypothesise that SpoIID and SpoIIP interact directly across the intermembrane space. This would imply that the role of SpoIIM is redundant in *C. difficile*, and suggests a SpoIID:SpoIIP complex rather than a DMP complex as is seen in *B. subtilis*.

The lack of membrane bulging in *C. difficile* Δ*spoIID*/Δ*spoIIP* mutants [21] suggests a possible secondary role in peptidoglycan remodelling beyond their enzymatic activity, involving recruitment and/or stabilisation of other proteins or other interactions with the cell wall and its components.

Elegant microscopy and complementation experiments on different combinations of multiple D/M/P/Q *C. difficile* mutants by Ribis *et al*. (2018) demonstrate that the DP and Q:AH machineries of *C. difficile* are able to complement each other to some extent. In Δ*spoIID*Δ*spoIIQ* double mutants, no heat-resistant spores are formed, though the severe membrane bulging morphological defects seen in the *spoIIQ* single mutant are reduced 5-fold. Furthermore, complementation of Δ*spoII*D*spoII*Q mutants with active SpoIID or SpoIIQ allows sporulation to continue in ∼0.05% of cells. This suggests some degree of functional redundancy in the roles of *Cd*Q and *Cd*IID [21].

Unlike in *B. subtilis* triple Δ*spoIID*Δ*spoIIP*Δ*spoIIM* mutant [27], a quadruple Δ*spoIID*Δ*spoIIP*Δ*spoIIM*Δ*spoIIQ* mutant did not generate a flat septum in *C. difficile*, though the reason for this continuing membrane curvature is unknown [21]. Perhaps in *C. difficile* there are unknown compensatory mechanisms that allow membrane curvature in the absence of DP/Q:AH mediated peptidoglycan degradation.

BACTH experiments have demonstrated that the DP and Q:AH machineries of *C. difficile* can interact directly, with strong interactions observed between SpoIID and SpoIIQ and between SpoIID and SpoIIIAH [15]. These same experiments showed only weak interactions between a catalytically inactive SpoIIP and SpoIID/M/Q and SpoIIIAH. Perhaps SpoIID is associated with SpoIIIAH in the mother cell membrane, whereas, as suggested by Ribis *et al*. (2018), SpoIIP is free in the intramembrane space and only forms weak or transient interactions with SpoIID/Q:AH. Alternatively, other anchoring or localisation mechanisms could be involved, perhaps via the SpoIIIAG ring or other proteins, that allow assembly of all the proteins required for engulfment. Co-localisation studies would help clarify the interplay between the different proteins.

Increasing awareness in the field that equivalent components of the engulfasome can fulfil different functions in *B. subtilis* and *C. difficile* has led to an increase in the number of studies conducted in *C. difficile*. However, many questions remain outstanding for a complete understanding of the engulfment driving forces in both organisms and the variations on engulfasome function and structure in *C. difficile*. Have all the proteins involved in the proposed engulfasome in *C. difficile* been identified? Are there specific PG synthases or modifying enzymes involved? Are the remaining *spoIIIA* operon proteins involved in engulfment? Where does SpoIIP localise *in vivo* and is the D(M)P machinery tethered or associated to the membrane? How is peptidoglycan digestion and synthesis coupled and what PG synthases are involved? What is the role of the DP/Q:AH assembly in spore coat localisation? Answering these questions will further our knowledge of engulfment and the basic biology of *C. difficile*. Such research also has the potential to reveal further therapeutic intervention possibilities, and, consequently, may lead to a reduction in the incidence of CDI and an improvement in patient outcomes.

## Conflicts of interest

The authors declare that they have no known competing financial interests or personal relationships that could have appeared to influence the work reported in this paper.
