# Supplementary material for: The engulfasome in C. difficile: Variations on protein machineries
Source: Anaerobe. 2019 Dec;60:102091. doi: 10.1016/j.anaerobe.2019.102091 (PMC6934232; doi:10.1016/j.anaerobe.2019.102091)
Supplement: Multimedia component 1 [file mmc1.pdf]

The engulfasome in *C. difficile*: variations on protein machineries

Supplementary Information

Abigail Kelly[1], Paula S. Salgado[1#]

[1]Institute for Cell and Molecular Biosciences, Faculty of Medical Sciences, Newcastle University, Newcastle upon Tyne, UK

[#] **Corresponding author**

Address: Institute for Cell and Molecular Biosciences, Faculty of Medical Sciences, Newcastle University, Newcastle upon Tyne, UK. Telephone: +44 (0)191 208 7432; paula.salgado@newcastle.ac.uk

Running title: *C. difficile* engulfasome overview

Keywords: *C. difficile*, *B. subtilis*, sporulation, engulfment, DMP, Q:AH

Conservation analysis – HMM searches

Protein sequences for SpoIIP, SpoIID and SpoIIM from *B. subtilis* (NP_390431.1, NP_391556.1 and NP_390234.1, respectively) and *C. difficile* (YP_001088983.1, YP_001086593.1 and YP_001087714.1, respectively) were used separately to search for orthologues amongst a set of 24 representative endosporeformers [1] using BLASTP [2]. Surprisingly, using CdSpoIIM retrieved only two orthologues during this search, despite the sequence conservation revealed in the sequence alignments (Fig. S1). A complete list of all orthologues was generated by combining the sequences retrieved in the BLAST searches and the Bacilli and Clostridia sequences were aligned separately using T-coffee [3]. This alignment was then used to produce a HMM profile in HMMBUILD [4] which was the basis for identifying orthologues of each protein in all Bacilli and all Clostridia as well as all bacteria with JackHMMER [5] within the HMMER3 web server [6]. The alignment of all Bacilli or all Clostridia SpoIID, SpoIIP and SpoIIM homologues retrieved from HMMER searches was then used to generate the final HMM profile from which motif conservation was analysed and weblogos created using Skylign [7]. As the alignment of all bacilli and all clostridial orthologues of SpoIID contained other LytB/PG hydrolysis protein families, as well as true SpoIID orthologues, we focused this particular conservation analysis on the alignment of the representative sporeformers after confirming domain architectures.

Signal peptide and transmembrane helix predictions

In order to investigate the presence of signal peptides in *B.subtilis* and *C. difficile* DMP, the proteins sequences were analysed using the SignalP 5.0 [8] and Phobius [9] servers. Similarly, the protein sequences were analysed in the TMHMM [10], Phobius and PSIPRED

[11] servers for identification of putative transmembrane helices. Following the standard used for UniProt annotations, a signal peptide was considered to be present if predicted by both SignalP 5.0 and Phobius and a transmembrane helix if predicted by TMHMM and Phobius. If the prediction of the signal peptide and transmembrane covered the same residues, Phobius was used to discriminate between the two possibilities, if necessary.

SpoIIP – representative Bacilli

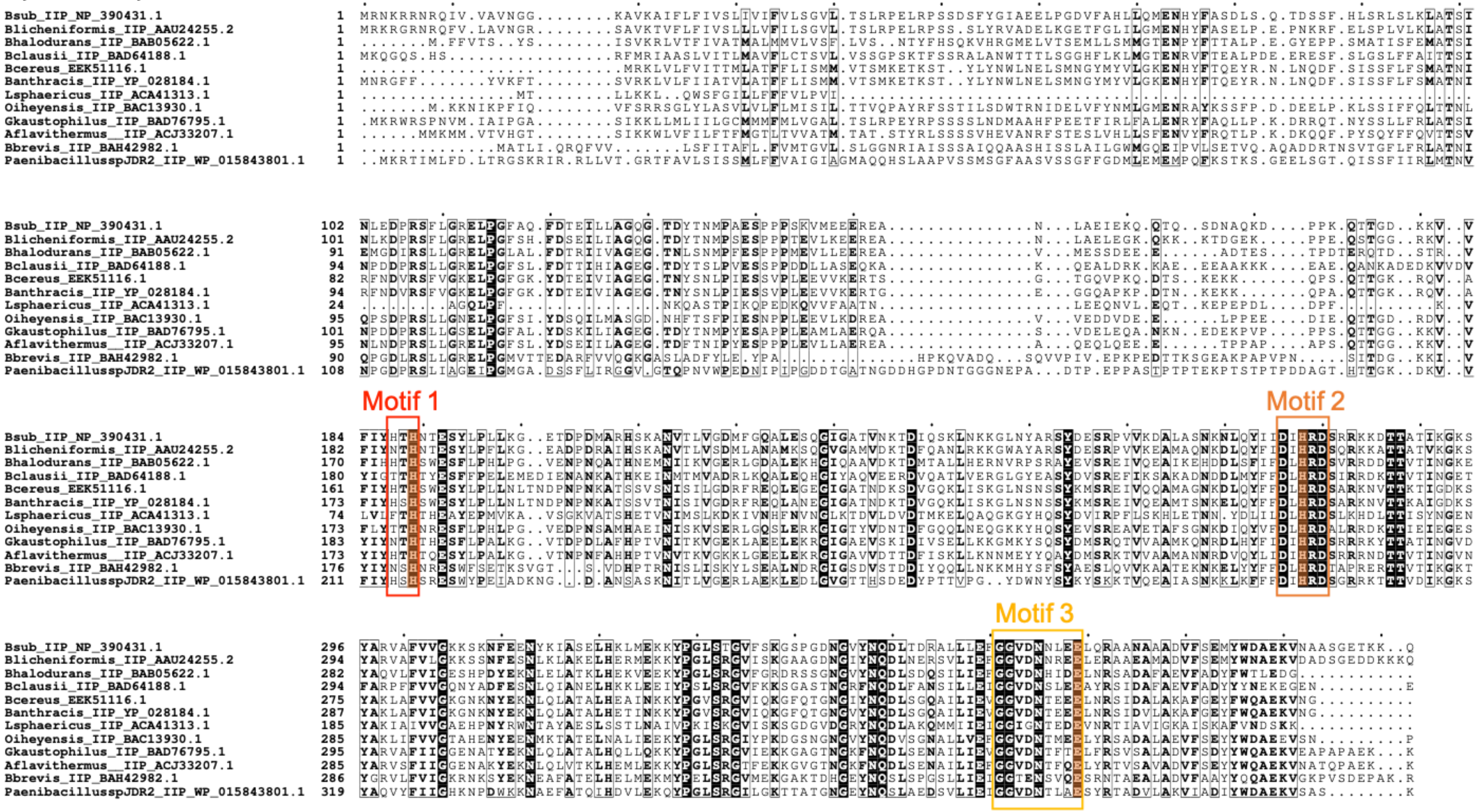

SpoIIP – representative Clostridia

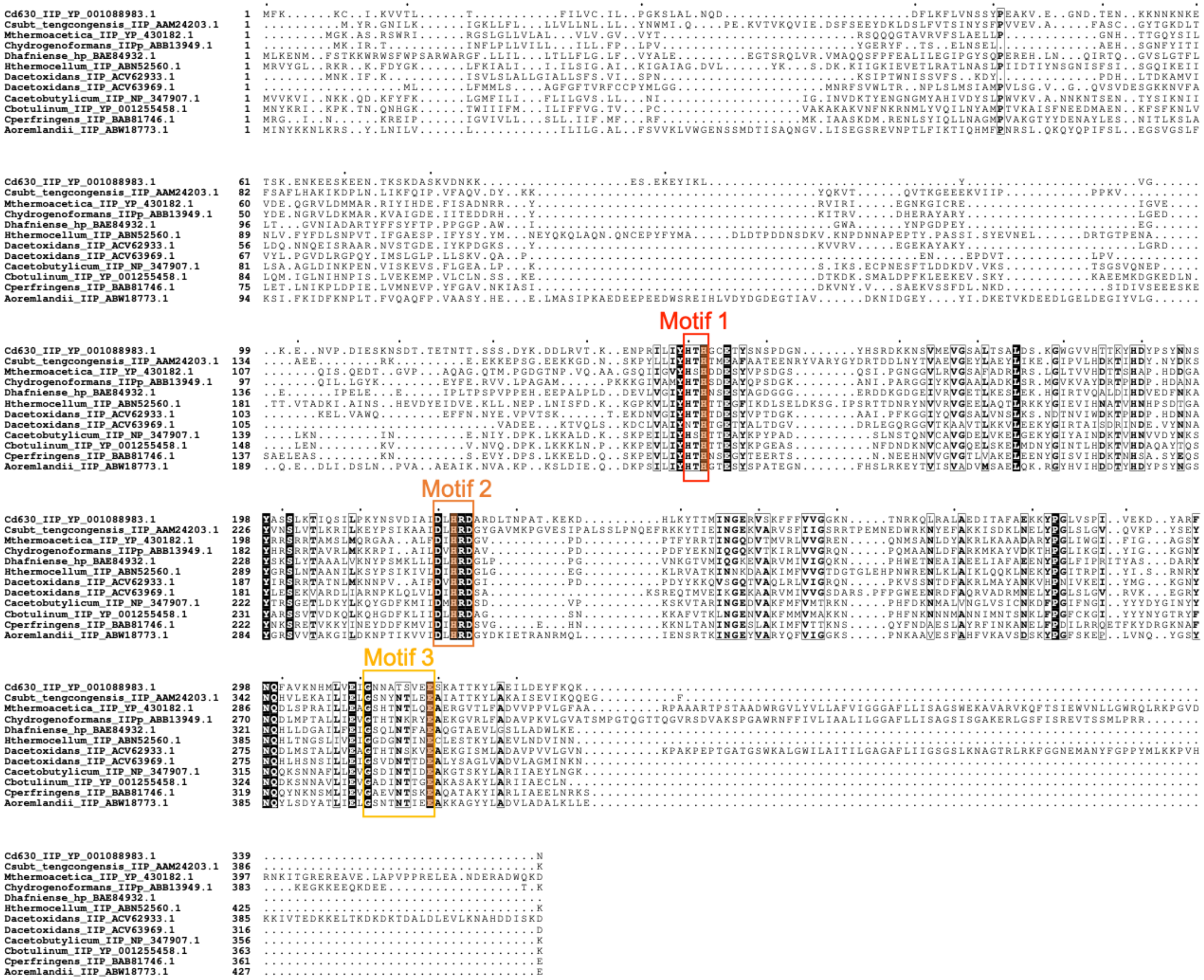

SpoIID – representative Clostridia

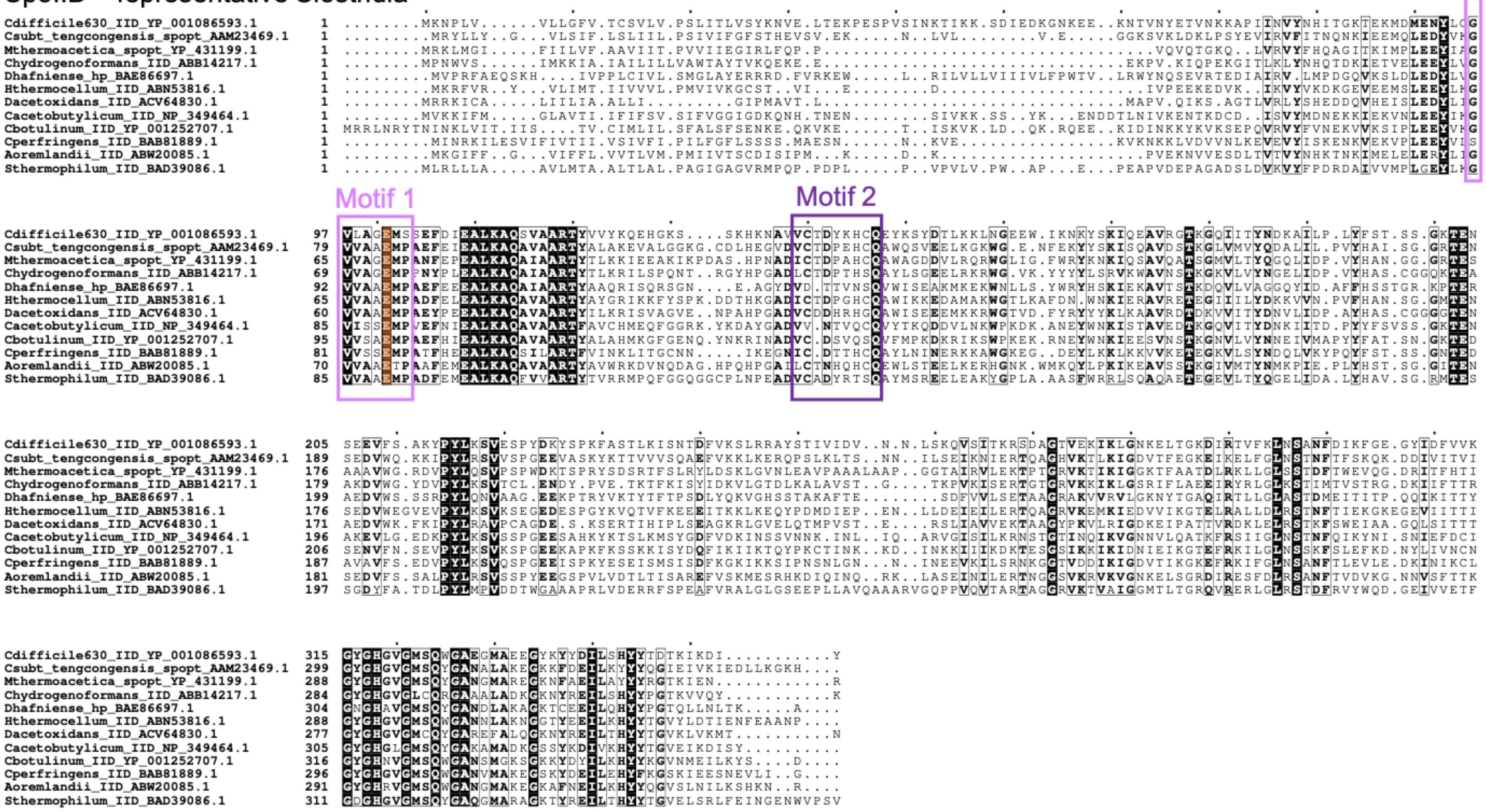

SpoIID – representative Bacilli

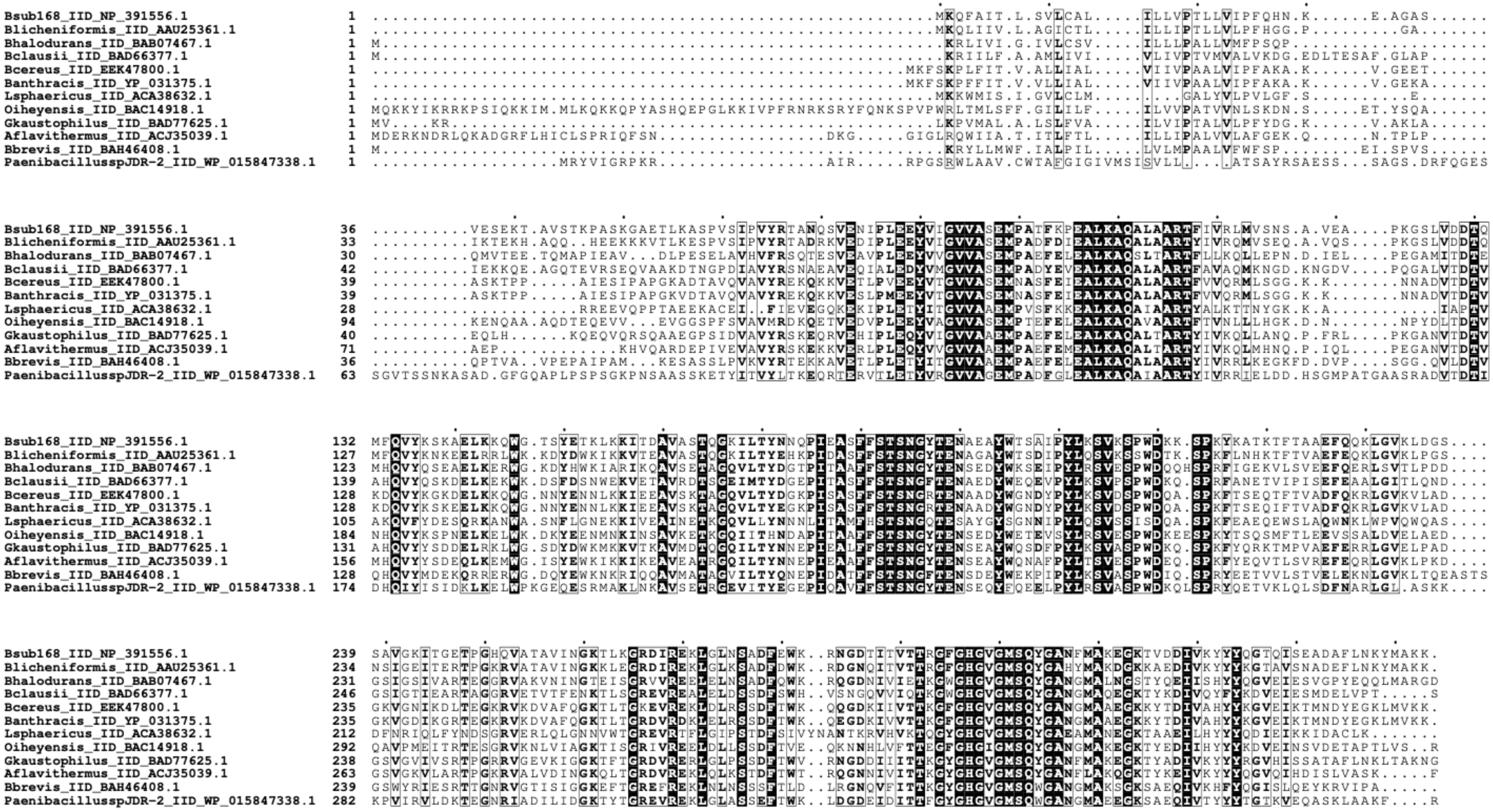

47

SpoIIM – representative Bacilli

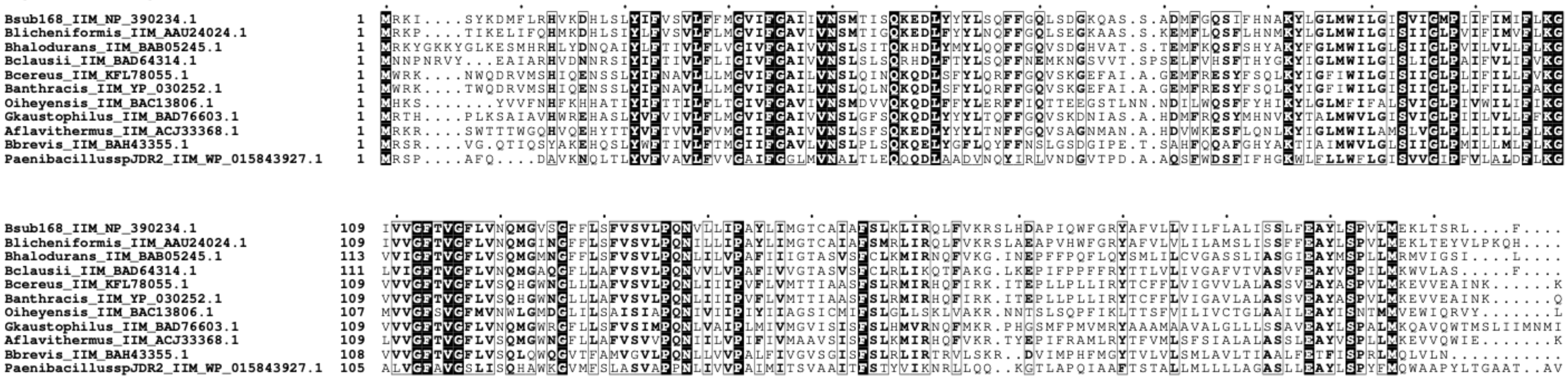

SpoIIM – representative Clostridia

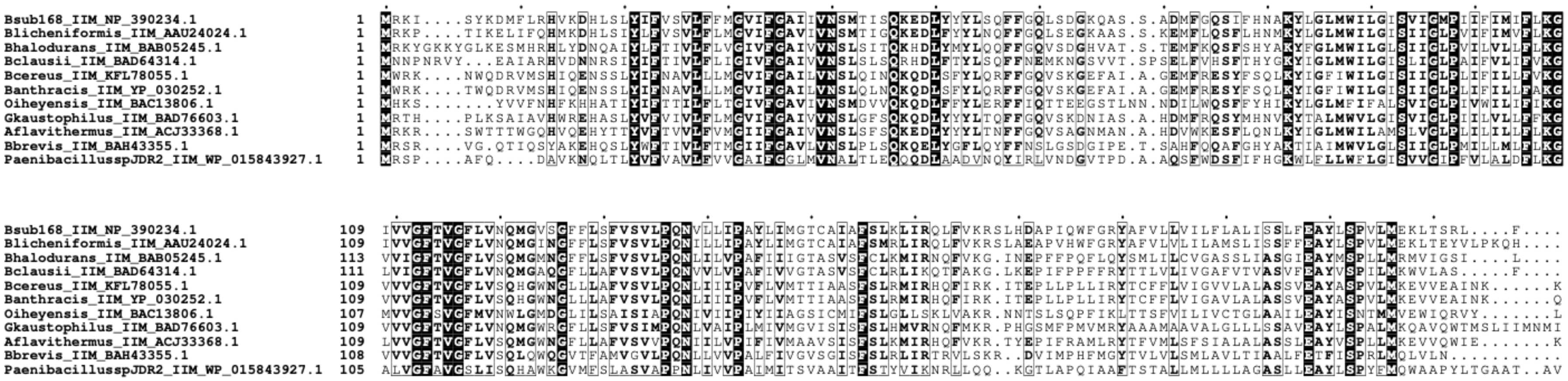

Fig. S1 – Multiple sequence alignment of DMP in representative endosporeformers

Protein sequences of SpoIIP, SpoIID and SpoIIM from *B. subtilis* and *C. difficile* were aligned with the sequences of retrieved orthologues in the representative Bacilli and Clostridia [1], respectively, using T-Coffee [3]. Motifs identified after full HMM searches in all Bacilli and Clostridia as well as the proposed catalytic residues are labelled: SpoIIP – motif 1, light purple; motif 2, dark purple; SpoIID – motif 1, red; motif 2, dark orange; motif 3, light orange. Catalytic residues in SpoIID and SpoIIP are shaded orange. Strictly conserved residues are boxed in a white background, residues with similarity score above 0.7 (ESPript) are shaded black. Alignment figures were generated with ESPript 3.0 [12].

[1] M.J.L. de Hoon, P. Eichenberger, D. Vitkup, Hierarchical evolution of the bacterial sporulation network., Curr. Biol. 20 (2010) R735-45. doi:10.1016/j.cub.2010.06.031.

[2] S.F. Altschul, W. Gish, W. Miller, E.W. Myers, D.J. Lipman, Basic local alignment search tool, J. Mol. Biol. 215 (1990) 403–410. doi:10.1016/S0022-2836(05)80360-2.

[3] C. Notredame, D.G. Higgins, J. Heringa, T-Coffee: A Novel Method for Fast and Accurate Multiple Sequence Alignment, J. Mol. Biol. 302 (2000) 205–217. doi:10.1006/jmbi.2000.4042.

[4] S.R. Eddy, Profile hidden Markov models, Bioinformatics. 14 (1998) 755–763. doi:10.1093/bioinformatics/14.9.755.

[5] L.S. Johnson, S.R. Eddy, E. Portugaly, Hidden Markov model speed heuristic and iterative HMM search procedure, BMC Bioinformatics. 11 (2010) 431. doi:10.1186/1471-2105-11-431.

[6] R.D. Finn, J. Clements, W. Arndt, B.L. Miller, T.J. Wheeler, F. Schreiber, A. Bateman, S.R. Eddy, HMMER web server: 2015 update, Nucleic Acids Res. 43 (2015) W30–W38. doi:10.1093/nar/gkv397.

[7] T.J. Wheeler, J. Clements, R.D. Finn, Skylign: a tool for creating informative, interactive logos representing sequence alignments and profile hidden Markov models, BMC Bioinformatics. 15 (2014) 7. doi:10.1186/1471-2105-15-7.

[8] J.J. Almagro Armenteros, K.D. Tsirigos, C.K. Sønderby, T.N. Petersen, O. Winther, S. Brunak, G. von Heijne, H. Nielsen, SignalP 5.0 improves signal peptide predictions using deep neural networks, Nat. Biotechnol. 37 (2019) 420–423. doi:10.1038/s41587-019-0036-z.

[9] L. Käll, A. Krogh, E.L.. Sonnhammer, A Combined Transmembrane Topology and Signal Peptide Prediction Method, J. Mol. Biol. 338 (2004) 1027–1036. doi:10.1016/J.JMB.2004.03.016.

[10] A. Krogh, B. Larsson, G. von Heijne, E.L.. Sonnhammer, Predicting transmembrane protein topology with a hidden markov model: application to complete genomes, J. Mol. Biol. 305 (2001) 567–580. doi:10.1006/JMBI.2000.4315.

[11] D.W.A. Buchan, D.T. Jones, The PSIPRED Protein Analysis Workbench: 20 years on, Nucleic Acids Res. (2019). doi:10.1093/nar/gkz297.

[12] X. Robert, P. Gouet, Deciphering key features in protein structures with the new ENDscript server, Nucleic Acids Res. 42 (2014) W320–W324. doi:10.1093/nar/gku316.

Table S1 – DMP orthologues identification in representative endosporeformers

	% identity to query					
	BsIID	CdIID	BsIIP	CdIIP	BsIIM	CdIIM
**Bacilli**						
Bacillus subtilis subsp. subtilis str. 168	-	35.7	-	29.4	-	
Bacillus licheniformis ATCC 14580	70.9	37.7	71.2		79.2	
Bacillus halodurans C-125	48.3	34.5	42.2		54.4	
Bacillus clausii KSM-K16	44.9	33.9	42.6		52.8	
Bacillus cereus ATCC 10987	38.5	42.7	45.8		51.9	
Bacillus anthracis str. Sterne	53.2	43.4	45.4		49.8	
Lysinibacillus sphaericus C3-41	38.5	34.9	30.0			
Oceanobacillus iheyensis HTE831	45.8	40.4	44.1		42.2	
Geobacillus kaustophilus HTA426	58.1	39.7	51.4	26.9	56.3	
Anoxybacillus flavithermus WK1	53.4	34.7	48.5	30.1	54.3	
Brevibacillus brevis NBRC 100599	46.5	32.6	37.0	27.1	48.0	
Paenibacillus sp. JDR-2	47.4	39.3	33.9	28.2	38.7	
**Clostridia**						
Caldanaerobacter subterraneus subsp. tengcongensis MB4	38.8	43.5	25.6	34.7	31.0	
Moorella thermoacetica ATCC 39073	44.0	37.7	31.3	29.6	39.6	
Carboxydothermus hydrogenoformans Z-2901	35.6	38.1		29.6	34.2	
Desulfitobacterium hafniense Y51	42.0	29.8	30.7	33.7	28.7	
Hungateilostridium thermocellum ATCC 27405	33.4	41.4		32.1	30.9	
Desulfotomaculum acetoxidans DSM 771	36.1	34.6	27.9	25.4	33.8	
Clostridium acetobutylicum ATCC 824	36.4	44.1	24.5	29.5	31.1	29.9
Clostridium botulinum A str. ATCC 3502	35.1	37.6	26.0	33.8	28.9	31.5
Clostridium perfringens str. 13	40.8	41.8	30.0	29.5	23.8	
Clostridium difficile 630	35.7	-	29.4	-		-
Alkaliphilus oremlandii OhILAs	37.9	40.8	33.2	37.9	31.2	
Symbiobacterium thermophilum IAM 14863	35.3	33.5			26.7	
